# First record of the New Guinea flatworm *Platydemus manokwari* (Platyhelminthes, Geoplanidae) as an alien species in Hong Kong Island, China

**DOI:** 10.3897/zookeys.873.36458

**Published:** 2019-08-29

**Authors:** Junjie Hu, Muhua Yang, Elysia Ruoyan Ye, Yulong Ye, Yao Niu

**Affiliations:** 1 School of Biological Sciences, Yunnan University, Kunming, 650091, China Yunnan University Kunming China; 2 Southeast Asia Biodiversity Research Institute, Chinese Academy of Science, Yezin, Nay Pyi Taw 05282, Myanmar Southeast Asia Biodiversity Research Institute, Chinese Academy of Science Nay Pyi Taw Myanmar; 3 St Joseph’s College, Hong Kong 999077, China St Joseph’s College Hong Kong China; 4 Chinese International School, Hong Kong 999077, China Hong Kong Youth Science Academy Hong Kong China; 5 Hong Kong Youth Science Academy, Hong Kong 999077, China Chinese International School Hong Kong China; 6 School of Life Science, Henan Normal University, Xinxiang 453007, China Henan Normal University Xinxiang China

**Keywords:** distribution, morphological characterisation, 18S rDNA, COX1

## Abstract

The New Guinea flatworm (*Platydemus manokwari*) caused extinction of the native land snails on several Pacific island in past decades, and therefore it has been listed among the top 100 of the world’s worst invasive alien species. Using morphological and molecular methods, New Guinea flatworms were discovered and identified for the first time in Hong Kong Island during a field investigation in July and August 2018. The flatworms were 32–60 mm long, 3–5 mm wide, and 1–2 mm thick. The dorsal side of the flatworm was dark brown with a thin yellow central line, and its ventral side appeared pale grey. To further verify this species, both 18S rDNA and mitochondrial cytochrome c oxidase subunit I gene (COX1) obtained from three specimens of *P. manokwari* were sequenced and analysed. While comparing these sequences with those previously deposited in GenBank, these 18S rDNA sequences shared 100% identity with the single available 18S rDNA sequence of *P. manokwari*; and the obtained COX1 sequences were identical to those of *P. manokwari* world genotype. Two native snails, *Criptosoma imperator* and *Bradybaena similaris*, have been found to be the prey of this predator during this investigation. Therefore, the invasive New Guinea flatworm certainly will cause a serious impact on the biodiversity of native snail populations, and an economic and environmental risk assessment for *P. manokwari* need to be completed in the near future in Hong Kong.

## Introduction

The land planarian *Platydemus manokwari* de Beauchamp, 1963, or New Guinea flatworm, is a highly invasive species, and the only flatworm listed in the top 100 of the world’s worst invasive alien species (Lowe et al. 2000). This species was first found on the New Guinea Island, but until now has been reported in 16 territories around the world (Justine et al. 2014, 2015, Chaisiri et al. 2018). The flatworms are carnivores, and feed upon a variety of soil organisms such as earthworms, isopods, insects, and snails, and may cause economic or environmental harm (Sugiura 2010, Justine et al. 2014). Additionally, they can harbour zoonotic pathogens which may possibly adversely affect human health (Chaisiri et al. 2018).

Morphological characteristics have traditionally been used as criteria to identify and distinguish different species of planarians. In the last decades, molecular methods have been used for delineation of marine, freshwater, and land planarians using different genetic markers (Carranza et al. 1998; Álvarez-Presas et al. 2008). To date, there are only one partial 18S rDNA sequence, one partial 28 rDNA sequence, and many partial mitochondrial cytochrome c oxidase subunit I gene (COX1) sequences obtained from limited isolates of *P. manokwari* available in GenBank. Interestingly, based on COX1, *P. manokwari* has been divided into two genetic haplotypes, “world” and “Australian” (Justine et al. 2015).

In the present study, New Guinea flatworms were discovered and identified based on the morphological and molecular characteristics in Hong Kong Island, China. To our knowledge, this is the first report of New Guinea flatworm in Hong Kong, even in China.

## Materials and methods

**Figure 1.**
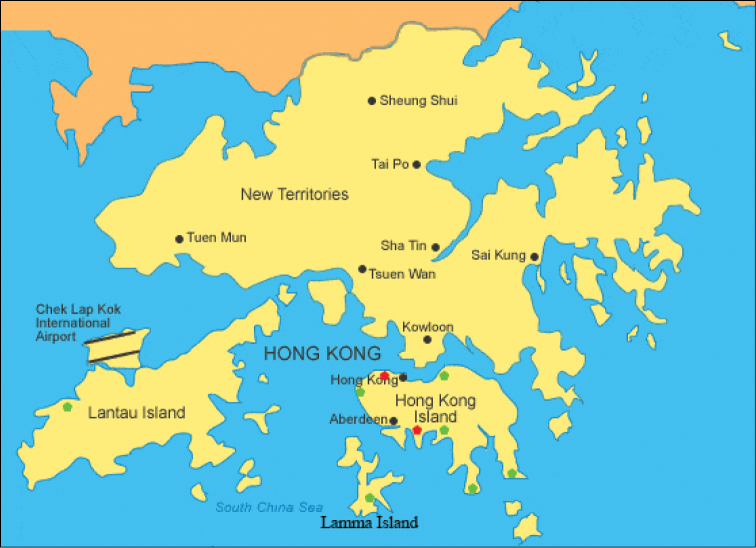
Localities of surveys for *Platydemus manokwari*. Green: not found; Red: found.

Hong Kong is a special administrative region of China and surrounded by the South China Sea on all sides except the north, which neighbours Shenzhen city along the Sham Chun River. The territory’s area consists of Hong Kong Island, the Kowloon Peninsula, the New Territories, Lantau Island, and more than 200 other islands. It has a humid subtropical climate, and approximately 40% of the remaining land area is devoted to country parks and nature reserves (Bernie and Raynor 2007). New Guinea flatworms were surveyed in nine localities (Fig. 1) including seven in Hong Kong Island, one in Lantau Island, and one in Lamma Island in July and August 2018. All these localities are along the coasts and covered by secondary forest. The flatworms were observed by the naked eye (32–60 mm long) and photographed using a Sony RX10 II camera.

For molecular identification, three specimens of *P. manokwari*, collected by hand and fixed in room-temperature ethanol (85%) were shipped to laboratory of zoology, Yunnan University, Kunming City, China. In the laboratory, a small piece of the body (ca. 4 mm^3^) was taken from the lateral edge of each ethanol-fixed individual. The remaining specimens were deposited at the Zoological Specimen Museum of Yunnan University (collection numbers Worm 2019011–13).

Genomic DNA was extracted using the phenol/chloroform method, after five rounds of freezing and thawing, and 0.01% proteinase K and 0.25% trypsin digestion. The 18S rDNA was amplified with primer pairs, 18SPmF/18SPmR (5'-ACCGCGGATGGCTCATTATA-3'/5'-ACGGAAACCTTGTTACGACTTTTA-3') designed using Premier 5 (Premier Biosoft International) based on the highly conserve regions of 18S rDNA sequences for members of Family Geoplanidae deposited in GenBank. The mitochondrial COX1 was amplified with primer pairs, BarS/ COI-ASmit2 (5'- GTTATGCCTGTAATGATTG-3'/ 5'-TAAAGAAAGAACATAATGAAAATG-3') designed by Álvarez-Presas et al. (2011) and Littlewood et al. (1997), respectively. Polymerase chain reaction (PCR) was performed in a total volume of 25 µl PCR cocktail that included 1X PCR buffer, 0.15 mmol MgCl_2_, 0.25 mmol dNTPs, 1 U Taq DNA polymerase (TakaRa, Dalian, China), 50–100 ng of DNA, and 25 pmol of each primer. For 18S rDNA, the amplification protocol was: 5 minutes at 94 °C, followed by 35 cycles of 95 °C for 1 minutes, 58 °C for 1 minutes, 72 °C for 2 minutes, with a final extension at 72 °C for 10 minutes. For mitochondrial *COX1*, PCR amplifications were performed as previously described (Justine et al. 2015). PCR products were gel purified and cloned to PMD-19T vector (TakaRa), and then sequenced on an ABI 3730XL automatic DNA sequencer (Applied Biosystems, Foster City, CA, USA). The sequences were assembled using multiple over lapping regions using the SeqMan II program (DNAStar, Madison, Wisconsin, USA), and then uploaded to GenBank and compared with other deposited sequences using the NCBI BLAST program.

## Results and discussion

**Figure 2.**
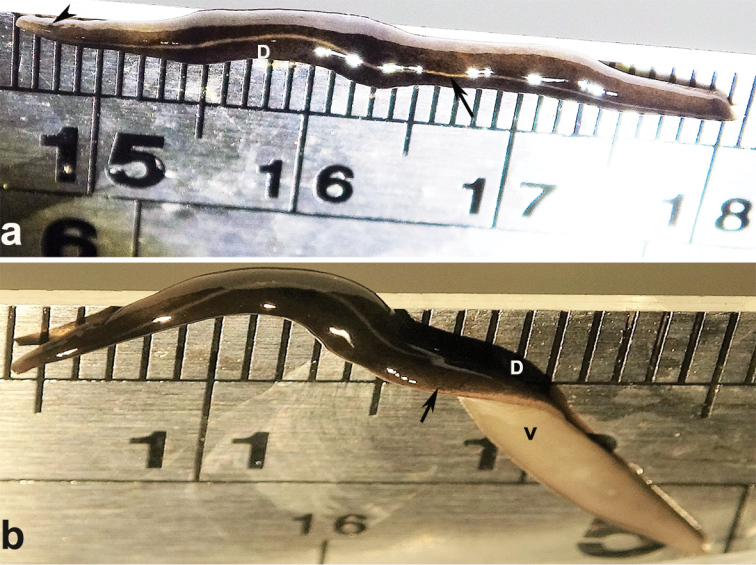
External morphological characteristics of *Platydemus manokwari*. Specimen collected in a dry ditch covered by dead leaves, Seavie Promenade, Hong Kong **A** dorsal view: note dorsal (D) with a median longitudinal line (arrow) and small eye situated back from the tip of the elongate snout-like head (arrowhead); **B** partial ventral view, showing the cream and faint grey marginal stripe (arrow) between dorsal (D) and ventral (V). Ruler in cm and mm.

New Guinea flatworms were only found in two of the nine localities, Seaview Promenade and Kennedy Road, both located in Hong Kong Island, Hong Kong. The habitats of this flatworm were in the deep mass of dead leaves. The flatworms were 32–60 mm long, 3–5 mm wide and 1–2mm thick (n = 12). The bodies were broadest in the middle, tapering at the both ends. Two small eyes were situated back from the tip of the elongate snout-like head (Fig. 2a). In cross section the flatworm was convex dorsally and flat ventrally. The dorsum was a dark olive brown colour, which under a lens showed a fine pale brownish graininess. A pale cream median dorsal longitudinal stripe, some 0.1 mm wide, began just behind the eyes and continued to the posterior tip (Fig. 2a). A thin sub-marginal cream stripe with fine lower greyish margin ran laterally from the anterior end along the length of the body (Fig. 2b). The ventral surface appeared off-white (Fig. 2b).

Genomic DNA was extracted from the three individual specimens of *P. manokwari*, respectively, and the 18S rDNA and mitochondrial COX1 were amplified successfully using their DNA as templates. The three 18S rDNA nucleotide sequences (MK959224–MK959226), each from an individual specimen of *P. manokwari* were 1697 bp in length, and completely identical. The most similar sequence in GenBank was that of *P. manokwari* (AF048766, 100% identity), followed by *Caenoplana* sp. (AJ270156, 94.7% identity). The three mitochondrial COX1 nucleotide sequences (MK959221–MK959223), also obtained from the three individuals were 948 bp in length and shared 100% identity. The most similar sequences in GenBank were those of *P. manokwari* world haplotype (100% identity) from Singapore (KR349579 and KR349580), the Solomon Islands (KR349586, KR349588-92), New Caledonia (KR349600-01, KT004666–KT004671), France (KR349594, KF887958), French Polynesia (KR349595), and USA (KR349610 and KR349611), followed by *P. manokwari* Australian haplotype (95.1–96.3% identity, on average 95.4 identity) from Australia (KR349581–KR349585, KF178320) and the Solomon Islands (KR349593, KR349602).

The external morphological characteristics of the specimens found in Hong Kong are similar to *P. manokwari* previously described from USA, Singapore, New Caledonia, French Polynesia, France, and Thailand (Justine et al. 2014, 2015; Chaisiri et al. 2018). Currently, *P. manokwari* has been recorded from 16 different territories, distributed in Australia, Asia, Europe, and central and South America, but mostly in the Indo-Pacific region where it originates (Justine et al. 2014, Chaisiri et al. 2018). As far as we are aware, this is the first report of New Guinea flatworms in Hong Kong, and therefore in China. This flatworm was introduced into Hong Kong probably through the international commercial trade of materials, particularly pottery and commercially and imported plants (either the soil or the plants themselves), which could be contaminated and easily transport the flatworm to new places (Sugiura 2009; Justine et al. 2014).

In our analysis, the new 18S rDNA sequences showed 100% identity among three specimens and completely identical to that of *P. manokwari* previously deposited in GenBank; the new mitochondrial COX1 sequences were also identical among different specimens, with 100% identity with those of *P. manokwari* world haplotype from different areas in GenBank. At mitochondrial COX1, *P. manokwari* has been divided into two haplotypes, “world” recorded from France, French Polynesia, Wallis and Futuna, New Caledonia, Singapore, Solomon Islands, USA, and Thailand; however, “Australian” only recorded those from the Solomon Islands and Australia (Justine et al. 2015; Chaisiri et al. 2018). The two haplotypes couldn’t be separated according to their external morphological characteristics (Justine et al. 2015), but the new obtained mitochondrial COX1 sequences of the world type only shared 95.4% identity with those of *P. manokwari* Australian type, and both invariably differed in 45 transitions/ substitutions of a 948-bp-long portion of the gene. Therefore, the “world type” and the “Australian type” represented a same or separate species within the genus *Platydemus* need more morphological and multi-genetic markers data to clarify their taxonomic positions in the future.

*Platydemus manokwari* has been recorded to feed mainly on land gastropod molluscs, but also on earthworms, insects, and nemerteans (Justine et al. 2014). During the present investigation, the native terrestrial molluscs, the arboreal snail (*Megaustenia imperator* Gould, 1859) and the Asian tramp snail (*Bradybaena similaris* Férussac, 1821) have been observed to be eaten by *P. manokwari* in the field. Therefore, the invasive New Guinea flatworm certainly will cause a serious impact on the biodiversity of native snail populations, and an economic and environmental risk assessment for *P. manokwari* need to be considered in the very near future.
